# Comparison of Different Local Anesthetic Volumes for Saddle Block Anesthesia in Ambulatory Surgery: A Prospective Randomized Trial

**DOI:** 10.7759/cureus.41063

**Published:** 2023-06-27

**Authors:** Emine N Zengin, Kudret Y Yalnız, Semih Başkan, Levent Öztürk

**Affiliations:** 1 Anesthesiology and Reanimation, Ankara Bilkent City Hospital, Ankara, TUR

**Keywords:** saddle block anesthesia, local anesthetic volume, discharge time, anorectal surgery, ambulatory surgery

## Abstract

Introduction: Saddle block anesthesia (SBA) is a frequently preferred method for ambulatory anorectal surgery. This study aimed to observe the effects of two different dose SBAs on discharge times and perioperative block characteristics in patients undergoing ambulatory anorectal surgery.

Methods: The study was conducted as a prospective, randomized controlled study. Patients over the age of 18 who were scheduled for ambulatory anorectal surgery and had American Society of Anaesthesiologists (ASA) physical status I and II were included in the research. Patients were divided into two groups: 5 mg hyperbaric bupivacaine 0.5% (Group I; n=34) and 3 mg hyperbaric bupivacaine 0.5% (Group II; n=34). The primary outcome was discharge time. Characteristics of the spinal block like time to reach S4 blockade, maximum blocked dermatome, regression time of sensorial, first analgesic need time, voiding time, mobilization time, and side effects were the secondary outcomes.

Results: Sixty-eight patients were included in the study. The groups were similar in terms of demographic and surgical characteristics (p > 0.05). In Group II, S4 sensory dermatome blockade time was statistically longer (p: 0.007) and the time to the disappearance of the sensory block was statistically shorter (p < 0.001). Also, voiding time and discharge times were statistically shorter in Group II (p: 0.049, p < 0.001, respectively).

Conclusion: SBA provided adequate anesthesia, and the complication rates were limited. Saddle block can be considered an advantageous technique because of conditions that adversely affect recoveries, such as postoperative cognitive problems, nausea, and vomiting due to general anesthesia. In addition, better recovery results and optimal surgical condition with 3 mg hyperbaric bupivacaine in our study suggest that this dose may be a good alternative.

## Introduction

Ambulatory surgery for anorectal disease includes a wide range of operations, including those for infectious issues such as abscesses, fistulas, and fulgurations as well as treatments for hemorrhoids and fissures. The current standard of care and customary practice allow for the completion of over 90% of these patients in an ambulatory environment. This ensures rapid discharge and over 90% patient satisfaction [1,2]. In addition, outpatient anorectal surgery is cost-effective as well as safe [3].

Although a deep plane of surgical anesthetic is required due to the anorectal region's dense sensory supply, these procedures are typically completed in a matter of minutes. During anorectal surgery, insufficient anesthetic has been linked to negative physiological reactions such as hypertension, tachycardia, and laryngospasm. In addition, if the postoperative pain cannot be prevented, it may prolong the patient's discharge time [4,5]. It might be difficult to manage the anesthesia for patients having ambulatory anorectal surgery [4].

Traditional anesthetic techniques for outpatient anorectal surgery may be compromised by a variety of issues, including prolonged onset, delayed offset, unreliability, and local anesthesia with intravenous sedation [6]. Saddle block anesthesia (SBA), a neuraxial technique, targets the distal sacral dermatomes to selectively block the perineum by intrathecal injection of local anesthetic while keeping the patient seated [4,7]. The spinal saddle anesthetic block has become the preferred method for perianal procedures, especially in high-volume day surgery facilities, because of additional characteristics such the quick onset, dense block, early patient ambulation, and a brief hospital stay [8].

Lower extremity motor strength preservation and a decrease in side effects including hypotension are claimed advantages over conventional spinal anesthesia. Furthermore, there is proof that SBA can boost operating room productivity, lower postoperative opioid usage, and raise general patient satisfaction [4,9]. Similar to spinal anesthesia, the kind and dosage of intrathecal local anesthetic given affects the block characteristics caused by SBA [4,10]. There is currently no agreement on the best local anesthetic regimen for SBA because of the diverse practice environment, substantial variation in the kinds and dosages of local anesthetic drugs utilized, and the relative scarcity of published information.

In this study, it was hypothesized that adequate anesthesia can be achieved with a lower local anesthetic volume for ambulatory surgery, as well as earlier discharge of the patients. Based on this hypothesis, this study aims to compare the effect of two different low-dose SBAs on perioperative block characteristics in patients undergoing ambulatory surgery.

## Materials and methods

This study was approved by the ethics committee of Ankara Bilkent City Hospital (ID: E1-22-2022; Date: 06.04.2022), and written informed consent was obtained from all subjects participating in the trial.

Patients over the age of 18 who were scheduled for ambulatory anorectal surgery and had American Society of Anaesthesiologists (ASA) physical status I and II were included in the research. Patients using chronic analgesic drugs, severe heart disease, diabetes, peripheral neuropathy, abnormal coagulation profiles, and obvious scoliosis were also excluded from the study.

Patients were divided into two groups (Group I and Group II) using a computer-generated randomization sequence. Group I (n=34) was performed 5 mg hyperbaric bupivacaine 0.5% (5 mg/mL, Bupivacaine, VEM Laboratories, Kapaklı/Tekirdağ, Türkiye) while Group II (n=34) was performed 3 mg hyperbaric bupivacaine 0.5% (5 mg/mL, Bupivacaine, VEM Laboratories).

Preoperative information was performed, including on instructions on postoperative wound care, pain management, preventing and managing constipation, activity limitations, and precautions. Patients were provided prescriptions for postoperative pain medication and stool softeners.

No patient was given premedication. In the operating room, patients were observed using electrocardiograms, non-invasive arterial blood pressure monitoring, and pulse oximetry. A 20 G cannula was placed on the back of the left hand and a 0.9% sodium chloride infusion of 8 mL/kg/hour was started. Spinal anesthesia was administered through the L4-5 or L5-S1 intervertebral space using a 25G Quincke-type spinal needle in the sitting position. Group I (n = 34) was performed 5 mg hyperbaric bupivacaine 0.5% while Group II (n = 34) was performed 3 mg hyperbaric bupivacaine 0.5% respectively. The test solution was injected slowly over one minute and the patients were held in a sitting position for five minutes to achieve adequate block. All spinal anesthesia applications were performed by a single experienced anesthesiologist.

Vital signs and anesthetic information were recorded by an anesthesiologist. Levels of sensory and motor blocks were assessed immediately before the jack-knife position. The motor block level was assessed using a modified Bromage scale [11] and the sensory level was assessed with a pinprick test. (0: no motor block; 1: can flex knee, move foot, but cannot raise leg; 2: can move foot only; 3: cannot move foot or knee). During surgery, in the post-anesthesia care unit, and in general wards, adverse effects from spinal anesthesia including hypotension, bradycardia, nausea, vomiting, and pruritus were noted. In addition, patients were called by phone after discharge and their pain status was followed up.

The primary outcome was discharge time. Characteristics of the spinal block like time to reach S4 blockade, maximum blocked dermatome, regression time of sensorial, first analgesic need time, voiding time, mobilization time, and side effects were the secondary outcomes. In addition, the patients were evaluated with the Modified Bromage Scale at the fifth minute after spinal anesthesia and for one hour (end of the surgery, postoperative 15th, 30th, 45th, and 60th minutes) after the surgery.

Statistical analysis

Data analysis was performed using the IBM SPSS 25.0 (IBM Corp., Armonk, NY, USA) statistical package program. While evaluating the study data, Chi-Square test was used to compare qualitative data as well as descriptive statistical methods (frequency, percentage, mean, standard deviation, median, min-max, interquartile range [IQR]). The conformity of the continuous data to the normal distribution was evaluated by Kolmogorov-Smirnow and Shapiro-Wilk tests, skewness-kurtosis and graphical methods (histogram, Q-Q Plot, Stem and Leaf, Boxplot). In the study, Independent Samples T-test (T-test in independent groups) was used for the comparison of quantitative data with normal distribution between groups, and Mann-Whitney U test was used for comparison between groups of data that did not show normal distribution. Statistical significance level was accepted as a=0.05.

Power analysis was carried out with the G*Power 3.1.9.7 (Franz Faul, Universitat Kiel, Kiel, Germany) statistical program package; n1 = 34, n2 = 34, α=0.05, Effect Size (d) = 1.0; power = 98% (Calculated by discharge time, the primary outcome).

## Results

The study was conducted between April 2022 and December 2022. A total of 68 patients, 18 female and 50 male, were included in the study. There was no statistically significant difference between the groups in terms of gender, age, height, weight, BMI, and ASA values of Group I and Group II patients (p > 0.05) (Table 1).

**Table 1:** Comparison of groups in terms of demographic characteristics ^a^: Chi-Square Test (n (%)), ^b^: Independent Samples T Test (Mean ± SD). ASA: American Society of Anesthesiologists, BMI: body mass index.

Patient characteristics		Group I (n=34)	Group II (n=34)	p
Gender (female)		7 (%20.6)	11 (%32.4)	0.410 ^a^
Age (year)		40.1 ± 14.6	41.5 ± 12.8	0.679 ^b^
Height (cm)		173.1 ± 7.4	168.6 ± 8.8	0.127 ^b^
Weight (kg)		82.4 ± 15.7	82.4 ± 14.9	0.994 ^b^
BMI (kg/m^2^)		27.4 ± 4.1	29.1 ± 5.7	0.159 ^b^
ASA	I	11 (%32.4)	19 (%55.9)	0.087 ^a^
	II	23 (%67.6)	15 (%44.1)	

The groups were statistically similar in terms of comorbidity (p > 0.05) (Table 2).

**Table 2:** Comparison of additional diseases between groups *: Chi-Square Test (n (%)). DM: diabetes mellitus, HT: hypertension, CAH: coronary artery disease, COPD: chronic obstructive pulmonary disease

Additional disease	Group I (n=34)	Group II (n=34)	p*
HT	2 (%5.9)	4 (%11.8)	0.673
DM	2 (%5.9)	1 (%2.9)	1.000
CAH	2 (%5.9)	--	0.493
COPD	1 (%2.9)	1 (%2.9)	1.000

It was determined that there was no statistically significant difference between the groups in terms of the type and duration of surgery (p > 0.05) (Table 3).

**Table 3:** Comparison of groups in terms of type and duration of surgery ^a^: Chi-Square Test (n (%)), ^b^: Independent Samples T Test (Mean ± SD)

Surgery	Group I (n=34)	Group II (n=34)	p
Pilonidal sinus excision	9 (%26.5)	4 (%11.8)	0.541^ a^
Anal fistulotomy	18 (%52.9)	21 (%61.8)	
Hemorrhoidectomy	3 (%8.8)	5 (%14.7)	
Rectal prolapse repair	1 (%2.9)	-	
Seton revision	1 (%2.9)	2 (%5.9)	
Anal condyloma excision	1 (%2.9)	-	
Anal polyp excision	1 (%2.9)	1 (%2.9)	
Anal fissure repair	-	1 (%2.9)	
Surgery time (minutes)	24.9 ± 10.8	22.9 ± 7.5	0.386^ b^

Hypotension, bradycardia, nausea, vomiting and pruritus were not observed in any of the patients.

It was observed that there was no statistically significant difference between the groups in terms of the time to delivery to surgery after spinal anesthesia and the need for sedation (p > 0.05). Sedation was required for three patients each in Group I and Group II (Table 4).

**Table 4:** Comparison of the groups in terms of delivery time to surgery and need for sedation after spinal anesthesia ^a^: Mann-Whitney U test (Median (Q1 – Q3)), ^b^: Chi-Square Test (n (%))

Duration and sedation	Group I (n=34)	Group II (n=34)	p
Delivery time to surgery after spinal anesthesia (minutes)	5.0 (4.8 – 6.0)	5.0 (5.0 – 6.0)	0.748^ a^
Need for sedation (yes)	3 (%8.8)	3 (%8.8)	1.000^ b^
2 mg midazolam + 50 mcg fentanyl	1 (%2.9)	2 (%5.9)	0.504^ b^
100 mg propofol + 50 mg ketamine	1 (%2.9)	--	
2 mg midazolam + 100 mcg fentanyl	--	1 (%2.9)	
50 mg ketamine + 50 mg propofol	1 (%2.9)	--	

While there was no statistically significant difference between the groups in terms of the highest dermatome reached by the block (p > 0.05), there was a statistically significant difference between the groups in terms of S4 sensory dermatome blockade time and time to disappearance of sensory block (p: 0.007, p < 0.001, respectively). In Group II, the duration of S4 sensory dermatome blockade was longer and the time to disappearance of sensory block was shorter (Table 5).

**Table 5:** Comparison of the groups in terms of S4 sensory dermatome blockage time – highest dermatome at which block occurs – disappearance time of sensory block ^a^: Independent Samples t Test (Mean ± SD), ^b^: Chi-Square Test (n (%))

Parameter	Group I (n=34)	Group II (n=34)	p
S4 sensory dermatome blockage time (minutes)	3.7 ± 1.5	4.5 ± 0.9	0.007^ a^
Highest dermatome at which block occurs			0.096^ b^
L1	2 (%5.9)	1 (%2.9)	
L5	-	2 (%5.9)	
S1	10 (%29.4)	7 (%20.6)	
S2	10 (%29.4)	4 (%11.8)	
S3	9 (%26.5)	9 (%26.5)	
S4	2 (%5.9)	10 (%29.4)	
S5	1 (%2.9)	1 (%2.9)	
Disappearance time of sensory block (hour)	3.7 ± 1.3	2.2 ± 0.5	<0.001^ a^

While there was no statistically significant difference between the groups in terms of first analgesic need times and mobilization times (p > 0.05), there was a statistically significant difference between the groups in terms of urination and discharge times (p: 0.049, p < 0.001, respectively). It was found that the urination and discharge times of Group II patients were shorter (Table 6).

**Table 6:** Comparison of the groups in terms of first analgesic need - voiding - mobilization and discharge times *: Independent Samples t Test (Mean ± SD)

Parameter	Group I (n=34)	Group II (n=34)	p*
First analgesic need time (hour)	4.5 ± 1.8	5.7 ± 2.8	0.144
Voiding time (hour)	3.6 ± 1.4	3.0 ± 0.9	0.049
Mobilization time (hour)	2.2 ± 1.0	2.0 ± 0.7	0.418
Discharge time (hour)	4.2 ± 0.9	3.3 ± 0.8	<0.001

The groups were similar at all times in terms of the Modified Bromage Scale (p > 0.05) (Table 7).

**Table 7:** Comparison of groups in terms of Modified Bromage Scale ^a^: Independent Samples T Test (Mean ± SD), ^b^: Chi-Square Test (n (%)). MBS: Modified Bromage Scale.

Modified Bromage Scale	Group I (n=34)	Group II (n=34)	p
5^th^ min after spinal anesthesia	0.00 ± 0.00	0.09 ± 0.29	0.083^ a^
Ankle, knee, and hip movable	34 (%100.0)	31 (%91.2)	0.239^ b^
Ankle and knee movable	-	3 (%8.8)	
End of the surgery	0.38 ± 0.70	0.26 ± 0.57	0.448^ a^
Ankle, knee, and hip movable	24 (%70.6)	27 (%79.4)	0.532^ b^
Ankle and knee movable	8 (%23.5)	5 (%14.7)	
Ankle movable only	1 (%2.9)	2 (%5.9)	
No movement	1 (%2.9)	-	
Postop 15^th^ min	0.38 ± 0.70	0.24 ± 0.55	0.339^ a^
Ankle, knee, and hip movable	24 (%70.6)	28 (%82.4)	0.396^ b^
Ankle and knee movable	8 (%23.5)	4 (%11.8)	
Ankle movable only	1 (%2.9)	2 (%5.9)	
No movement	1 (%2.9)	-	
Postop 30^th^ min	0.35 ± 0.69	0.24 ± 0.55	0.441^ a^
Ankle, knee, and hip movable	25 (%73.5)	28 (%82.4)	0.508^ b^
Ankle and knee movable	7 (%20.6)	4 (%11.8)	
Ankle movable only	1 (%2.9)	2 (%5.9)	
No movement	1 (%2.9)	-	
Postop 45^th^ min	0.29 ± 0.63	0.18 ± 0.46	0.381^ a^
Ankle, knee, and hip movable	26 (%76.5)	29 (%85.3)	0.394^ b^
Ankle and knee movable	7 (%20.6)	4 (%11.8)	
Ankle movable only	-	1 (%2.9)	
No movement	1 (%2.9)	-	
Postop 60^th^ min	0.24 ± 0.61	0.12 ± 0.33	0.324^ a^
Ankle, knee, and hip movable	28 (%82.4)	30 (%88.2)	0.554^ b^
Ankle and knee movable	5 (%14.7)	4 (%11.8)	
No movement	1 (%2.9)	-	
Time to zero of MBS (hour) (10/7)	1.65 ± 0.85	1.11 ± 0.61	0.170^ a^

## Discussion

The results of this study, in which two different doses of spinal hyperbaric bupivacaine were administered for saddle block, showed that although similar results were obtained in terms of application in general, rapid regression of the block was observed in the low-dose hyperbaric bupivacaine group. However, the time for full activation of spinal anesthesia was longer. Voiding time and discharge time were shorter in the low-dose bupivacaine group.

With the advantages it provides and significantly reducing the hospital burden, outpatient surgery clinical applications have increased gradually. For this reason, they have become significant applications of health centers. Since the operations performed are usually uncomplicated and sometimes of very short duration, day surgery and discharge on the same day are appropriate. However, while most implementations are short, a comprehensive block implementation is sometimes unavoidable in most cases. Some of these surgeries are anorectal surgeries, as in our study [4].

Although spinal anesthesia is becoming increasingly popular for ambulatory surgery, until recently, its use in ambulatory surgery was limited due to the lack of a safe, licensed, short-acting local anesthetic agent. An ideal intrathecal agent for ambulatory surgery should have a rapid onset of motor and sensory blockade, predictable regression within an acceptable time frame, and a low incidence of adverse events [12,13]. On the other hand, its important advantages are eliminating the need for airway manipulation, reducing the need for hypnotic sedatives, and reducing the side effects associated with muscle relaxants. This may be particularly beneficial in high-risk patients, such as the elderly, who may be at high risk for postoperative cognitive decline. Similarly, patients with comorbid conditions such as obesity and obstructive sleep apnea may benefit from avoiding airway instrumentation and the effects of anesthetic drugs used for general anesthesia. In addition, the residual analgesia provided by spinal anesthesia postoperatively may limit opioid use. This is associated with lower postoperative pain scores and opioid requirements in spinal anesthesia [14].

Although the advantages and disadvantages of general anesthesia and spinal anesthesia applications in ambulatory surgery are still an ongoing debate, the saddle block applied in anorectal surgery can be considered an attractive anesthesia method due to its very limited area of action. Studies show that the saddle block applied in anorectal surgery is an advantage as it provides adequate surgical anesthesia and limits the side effects associated with general anesthesia [7]. Patterson et al. [7] provided satisfactory surgical anesthesia conditions in a study in which they analyzed the data of patients who applied hyperbaric bupivacaine or ropivacaine for anorectal surgery. This study found that the average outpatient stay at the center was three hours and 53 minutes, while the incidence of side effects was quite low. In the present study, the absence of overnight hospitalization and the very limited complication rate indicate that the saddle block can be used safely in outpatient anorectal surgery. In addition, the limited need for sedation in these patients is important in terms of preventing the prolonged hospitalization period caused by sedative agents.

One of the most important goals of day surgery is to provide safe and effective anesthesia and to ensure that patients are safely discharged as soon as possible by limiting anesthesia-related side effects. For this purpose, determining the optimal anesthesia method and creating effective anesthesia using the lowest possible dose of anesthetic are key factors. However, the issue of how to determine the optimal anesthetic dose is still one of the most important questions. Different local anesthetics in different volumes are used in the saddle block, which is stated to be safe to be used in studies [4,7,15]. Patterson et al. [7] used 2.5-5 mg of hyperbaric local anesthetic in a large-series cohort study they conducted and stated that an analgesic effect was achieved for one to three hours depending on the half-life of the local anesthetic. Yung et al. [4] also evaluated 11 studies in systemic review analysis, and dose preferences ranged from 1.5-7.5 mg for short-acting local anesthetics and 3-30 mg for long-acting local anesthetics. The study also found that the median time to discharge was similar across all subgroups, at 182 minutes. The use of long-acting, lower-dose regimens was associated with a faster median time to motor block regression. In the present study, adequate analgesia was provided for surgery in both groups, and the block effect ended in 132 minutes in the group administered 3 mg hyperbaric bupivacaine. This shows that the recovery time is quite fast, and the provision of adequate analgesia in patients also allows safe surgical application. In addition, urinary retention, which is one of the biggest problems limiting spinal anesthesia applications, was absent in both groups, and the first voiding time was faster in the group administered 3 mg local anesthetic. Similar sedation requirements and short recovery suggest that 3 mg hyperbaric bupivacaine can be used safely for saddle block in anorectal surgeries.

There are some limitations in this study. First, the study is single-center. This may limit the generalization of results to the population. Second, the sample size is limited. Third, the study was evaluated using only two different doses. In addition, post-spinal headaches and other possible anesthesia-related complications after discharge were not evaluated.

## Conclusions

In conclusion, there is still no clear consensus on the anesthesia method to be chosen in outpatient anorectal surgery. Spinal anesthesia, and especially SBA, provides adequate anesthesia, and complications are limited. Saddle block can be considered an advantageous technique because of conditions that adversely affect recoveries, such as postoperative cognitive problems, nausea, and vomiting due to general anesthesia. In addition, better recovery results and optimal surgical condition with 3 mg hyperbaric bupivacaine in our study suggest that this dose may be a good alternative. Large series of controlled randomized studies with different dosing regimens will help to clarify this issue.
